# Vaccine Safety and Immunogenicity in Patients With Multiple Sclerosis Treated With Natalizumab

**DOI:** 10.1001/jamanetworkopen.2024.6345

**Published:** 2024-04-12

**Authors:** René Carvajal, Ana Zabalza, Pere Carbonell-Mirabent, Xavier Martínez-Gómez, Juliana Esperalba, Agustín Pappolla, Ariadna Rando, Alvaro Cobo-Calvo, Carmen Tur, Marta Rodriguez, Jordi Río, Manuel Comabella, Joaquín Castilló, José Ángel Rodrigo-Pendás, Nathane Braga, Neus Mongay-Ochoa, Claudia Guío-Sánchez, Ángela Vidal-Jordana, Georgina Arrambide, Breogán Rodríguez-Acevedo, Luciana Midaglia, Blanca Borras-Bermejo, Ingrid Galán, Jaume Sastre-Garriga, Xavier Montalban, Susana Otero-Romero, Mar Tintoré

**Affiliations:** 1 Department of Neurology-Neuroimmunology, Multiple Sclerosis Centre of Catalonia (Cemcat), Hospital Universitari Vall d’Hebron, Universitat Autònoma de Barcelona, Barcelona, Spain; 2 Department of Preventive Medicine and Epidemiology, Hospital Universitari Vall d’Hebron, Universitat Autònoma de Barcelona, Barcelona, Spain; 3 Department of Microbiology, Hospital Universitari Vall d’Hebron, Universitat Autònoma de Barcelona, Barcelona, Spain; 4 CIBER de Enfermedades Infecciosas (CIBERINFEC), Instituto de Salud Carlos III, Madrid, Spain; 5 Universitat de Vic-Universitat Central de Catalunya (UVic-UCC)

## Abstract

**Importance:**

Vaccination in patients with highly active multiple sclerosis (MS) requiring prompt treatment initiation may result in impaired vaccine responses and/or treatment delay.

**Objective:**

To assess the immunogenicity and safety of inactivated vaccines administered during natalizumab treatment.

**Design, Setting, and Participants:**

This self-controlled, prospective cohort study followed adult patients with MS from 1 study center in Spain from September 2016 to February 2022. Eligible participants included adults with MS who completed immunization for hepatitis B virus (HBV), hepatitis A virus (HAV), and COVID-19 during natalizumab therapy. Data analysis was conducted from November 2022 to February 2023.

**Exposures:**

Patients were categorized according to their time receiving natalizumab treatment at the time of vaccine administration as short-term (≤1 year) or long-term (>1 year).

**Main Outcomes and Measures:**

Demographic, clinical, and radiological characteristics were collected during the year before vaccination (prevaccination period) and the year after vaccination (postvaccination period). Seroprotection rates and postvaccination immunoglobulin G titers were determined for each vaccine within both periods. Additionally, differences in annualized relapse rate (ARR), new T2 lesions (NT2L), Expanded Disability Status Scale (EDSS) scores, and John Cunningham virus (JCV) serostatus between the 2 periods were assessed.

**Results:**

Sixty patients with MS (mean [SD] age, 43.2 [9.4] years; 44 female [73.3%]; 16 male [26.7%]; mean [SD] disease duration, 17.0 [8.7] years) completed HBV, HAV, and mRNA COVID-19 immunization during natalizumab treatment, with 12 patients in the short-term group and 48 patients in the long-term group. The global seroprotection rate was 93% (95% CI, 86%-98%), with individual vaccine rates of 92% for HAV (95% CI, 73%-99%), 93% for HBV (95% CI, 76%-99%), and 100% for the COVID-19 messenger RNA vaccine (95% CI, 84%-100%). Between the prevaccination and postvaccination periods there was a significant reduction in the mean (SD) ARR (0.28 [0.66] vs 0.01 [0.12]; *P* = .004) and median (IQR) NT2L (5.00 [2.00-10.00] vs 0.81 [0.00-0.50]; *P* = .01). No changes in disability accumulation were detected (median [IQR] EDSS score 3.5 [2.0-6.0] vs 3.5 [2.0-6.0]; *P* = .62). No differences in safety and immunogenicity were observed for all vaccines concerning the duration of natalizumab treatment.

**Conclusions and Relevance:**

The findings of this cohort study suggest that immunization with inactivated vaccines during natalizumab therapy was both safe and immunogenic, regardless of the treatment duration. Natalizumab may be a valuable option for proper immunization, averting treatment delays in patients with highly active MS; however, this strategy needs to be formally evaluated.

## Key Points

**Question:**

Are inactivated vaccines safe and immunogenic in patients with multiple sclerosis (MS) who are treated with natalizumab?

**Findings:**

In this cohort study of 60 patients with MS treated with natalizumab, seroprotection rates exceeded 90% for hepatitis A, hepatitis B, and/or messenger RNA COVID-19 vaccines. Additionally, all vaccines exhibited a favorable safety profile, indicating no disease activity exacerbation.

**Meaning:**

These findings suggest that inactivated vaccines administered during natalizumab treatment are safe and immunogenic, offering a valuable option for patients with highly active MS, averting treatment delays.

## Teaser

This cohort study assesses the immunogenicity and safety of inactivated vaccines for patients with multiple sclerosis (MS) in Spain receiving natalizumab treatment.

## Introduction

In recent years, there has been a growing availability of high-efficacy therapies for the treatment multiple sclerosis (MS).^1,2^ These treatments may increase the risk of acquiring new infections, reactivating latent pathogens, or worsening ongoing infectious conditions.^3^ In this context, current therapeutic strategies for MS should carefully consider the infectious risks associated with each treatment and incorporate risk mitigation measures, such as vaccination.^4,5,6^ However, the immunogenicity of vaccination can be compromised by immunosuppressive agents, particularly anti-CD20 therapies that are currently widely used.^7^ Consequently, clinicians eventually opt to delay the initiation of such therapies until vaccination schedules are completed, which could potentially have unfavorable consequences on the prognosis of the disease.^8,9^ Conversely, rapidly initiating anti-CD20 therapy without prior vaccination could potentially expose nonimmune people with MS (PWMS) to severe, vaccine-preventable infections.

Natalizumab, a monoclonal antibody targeting α-4 integrin, is a highly effective therapy that appears to preserve immune responses to both naive and recall antigens.^10,11,12,13,14,15,16^ Nevertheless, existing research on vaccine responses in patients treated with natalizumab predominantly focuses on respiratory viruses like influenza, providing limited data on other commonly administered vaccines.^11^ Additionally, most of these studies^12^ have generally evaluated immune responses in patients using various disease-modifying therapies (DMTs), which involved only a small subgroup of patients receiving natalizumab.

Lastly, the few reports of disease worsening following vaccination have raised controversy around vaccine safety.^17,18,19^ This issue is particularly crucial for those with highly active MS because there is a scarcity of available data in this specific population. The present study aims to assess the immunogenicity and safety of inactivated vaccines for hepatitis B virus (HBV), hepatitis A virus (HAV), and messenger RNA (mRNA) COVID-19 in patients with highly active MS undergoing treatment with natalizumab in an clinical setting.

## Methods

### Patient Selection and Study Design

This cohort study was approved by the clinical research ethics committee at Vall d’Hebron University Hospital and followed the Strengthening the Reporting of Observational Studies in Epidemiology (STROBE) reporting guideline. This study was conducted using data from a cohort of patients resulting from the merger of 2 ongoing prospectively followed cohorts^20,21^ initiated in 1995 at the Multiple Sclerosis Centre of Catalonia (Cemcat) in Spain. The first cohort,^20^ known as the Barcelona Clinically Isolated Syndromes (CIS) Inception Cohort, comprises adult PWMS aged younger than 50 years who experienced a CIS within 3 months of their initial clinical assessment. The second cohort,^21^ referred to as the Barcelona Treatment Cohort, includes patients currently being monitored since the initiation of any available DMT. Demographic, clinical, radiological, and biological data were systematically collected for all patients following a standardized protocol which included regular clinical assessments for relapses, evaluations of the Expanded Disability Status Scale (EDSS), and magnetic resonance imaging (MRI) scans (all routinely conducted by neuroradiologists). Databases have been developed according to national and international standards on ethical aspects (Declaration of Helsinki^22^ and Declaration of Tokyo^23^). All patients signed written informed consent according to the Declaration of Helsinki.^22^

### MRI Data Acquisition

MRI scans were performed on 1.5 T or 3.0 T superconductive magnets using a standardized protocol that included the following sequences: (1) transverse 2-dimensional (2D) dual-echo, T2-weighted fast spin echo; (2) transverse 2D, T2-weighted, fluid-attenuated inversion-recovery; and (3) transverse 2D T1-weighted, spin-echo or gradient-echo. For all sequences, 46 interleaved contiguous axial sections were acquired with a 3-mm section thickness covering the whole brain and an in-plane spatial resolution of approximately 1 × 1 mm. The transverse T1-weighted sequence after a gadolinium-based contrast agent administration was not routinely performed for all patients because this is not a standardized protocol at our institution for patients receiving natalizumab. The number of T2 lesions at baseline MRI and the number of new T2-weighted lesions (NT2L) on follow-up scans were assessed visually. A single experienced rater, blinded to patient clinical data and with expertise in inflammatory demyelinating diseases, performed the MRI analysis procedure.

### Vaccination Immunogenicity Assessment

Starting in 2015, PWMS at Cemcat were routinely referred to the preventive medicine department at Vall d’Hebron University Hospital for a baseline serostatus evaluation and immunization as per current guidelines.^24,25^ Postvaccination serological tests were consistently carried out within 1 to 3 months after vaccination. For the purposes of this study, we enrolled patients who received at least 1 of the following vaccinations between September 2016 and February 2022: HAV, HBV (enhanced immunity high load or adjuvanted), or COVID-19 (BNT162b2 [Pfizer-BioNTech], mRAN-1273 [Moderna], or ChAdOx1-S [recombinant; AstraZeneca]). The criteria for vaccination against HBV and HAV involved individuals having baseline serostatus evaluation with antibody titers below the accepted cutoff for protection. As for BNT162b2, mRNA-1273, and ChAdOx1-S, patients received the vaccine in accordance with the prevailing national immunization guidelines at the time of the study.^26^ Patients were required to complete the immunization schedule while receiving natalizumab treatment and have an available postvaccination serological test, which covered HAV immunoglobulin G (IgG) antibody (anti-HAV; Siemens Atellica IM hepatitis A total), hepatitis B surface antibody (anti-HBs; Roche Elecsys anti-HBs II), and SARS-CoV-2 spike-protein IgG antibody (antispike; Roche Elecsys anti–SARS-CoV-2). Seroprotection rates were defined as the percentage of patients achieving an adequate humoral response in the postvaccination serostatus evaluation, based on the accepted cutoff levels for each vaccine (anti-HAV, ≥0.02 mIU/L [to convert to IU/L, multiply by 1000]; anti-HBs, ≥0.01 mIU/L; and antispike, ≥13.0 antibody units/mL).^27,28,29^ Furthermore, postvaccination IgG titers were also evaluated.

### Vaccination Safety Assessment

We conducted a retrospective, self-control analysis, comparing the annualized relapse rate (ARR), EDSS score, and NT2L counts during the 12 months prior to vaccination (prevaccination period) with those in the 12 months following vaccination (postvaccination period). We designated the first trimester following vaccination as the high-risk period for potential disease exacerbation, consistent with previous studies.^30,31^ Furthermore, patients were categorized on the basis of their duration of natalizumab treatment before vaccination: short-term when administered within the first year of natalizumab initiation and long-term when administered after the first year of natalizumab treatment (eFigure in Supplement 1).

### JC Virus Status

The John Cunningham virus (JCV) serostatus of each patient was assessed both before and during natalizumab treatment, following current guidelines.^32^ If patients experienced an increase in their JCV index or treatment failure necessitating a change in their treatment plan, these cases were managed based on the clinical judgment of the treating neurologists.

### Statistical Analysis

Because all eligible patients were included, a specific sample size calculation was not conducted. Descriptive statistics were used to assess demographic and clinical data. Continuous variables were expressed as mean (SD) and median (IQR) while categorical variables were presented as percentages. To compare groups, χ^2^ tests were used for qualitative variables and Student *t *or Mann Whitney *U *tests were applied for quantitative variables, as appropriate. We further examined clinical and paraclinical characteristics between the prevaccination and postvaccination periods, depending on the duration of natalizumab treatment before vaccination (long-term vs short-term), using appropriate parametric or nonparametric tests.

Seroprotection rates, along with their 95% CIs, were determined for each vaccine. All statistical analyses were carried out using R statistical software version 4.2.2 (R Project for Statistical Computing). Data analysis was conducted from November 2022 to February 2023.

## Results

**Figure 1.**
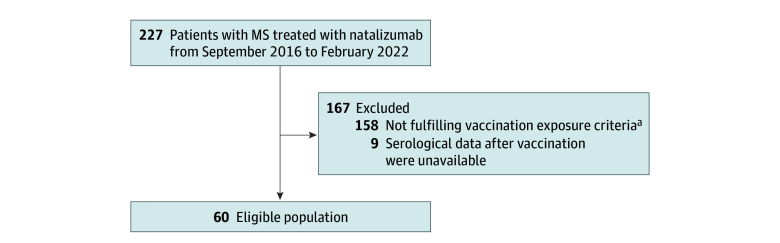
Patient Flow Diagram MS indicates multiple sclerosis. ^a^Criteria included at least the first 3 doses of the hepatitis B primovaccination schedule (0, 1, 2, and 6-12 months); at least the first dose of the hepatitis A vaccination schedule (0 and 6-12 months); 2 doses of the COVID-19 primovaccination schedule (BNT162b2 and mRNA-1273, 0 and 1 months; ChAdOx1-S, 0 and 3 months).

Sixty patients from the Barcelona CIS Inception and Treatment cohorts (mean [SD] age, 43.2 [9.4] years; 44 female [73.3%]; 16 male [26.7%]; mean [SD] disease duration, 17.0 [8.7] years) completed vaccine schedules while undergoing natalizumab treatment and were included in the analysis, with 12 patients in the short-term group and 48 patients in the long-term group (Figure 1). Among all 60 patients, 21 (35.0%) received the HAV vaccine, 27 (45.0%) received the HBV vaccine, and 23 (38.3%) received the mRNA COVID-19 vaccine. Thirty patients (50.0%) transitioned to anti-CD20 therapy after immunization due to high JCV index and/or disease progression. Demographic and clinical characteristics at the time of vaccination between the short-term and long-term groups can be found in the Table. Compared with PWMS treated with natalizumab in the inclusion period who were not vaccinated (167 patients), vaccinated patients were older (mean [SD] age, 45.1 [9.4] vs 41.0 [10.6] years; *P* = .01), had a longer mean (SD) duration of natalizumab treatment (97.1 [53.9] vs 72.3 [56.7] months; *P* = .003), and had a longer mean (SD) disease duration (19.2 [8.9] vs 15.9 [8.7] years; *P* < .001) (eTable 1 in Supplement 1).

**Table.** Demographic and Clinical Characteristics at the Time of Vaccination in the Short-Term and Long-Term Groups

General characteristics	Participants by duration of natalizumab treatment, No. (%)			*P* value
	Overall (N = 60)	Short-term (n = 12)	Long-term (n = 48)	
Age at vaccination, mean (SD), y	43.2 (9.4)	39.1 (6.3)	44.2 (9.8)	.09
Sex				
Female	44 (73.3)	8 (66.6)	36 (75.0)	.71
Male	16 (26.7)	4 (33.4)	12 (25.0)	.71
Change natalizumab to anti-CD20 after vaccination	30 (50.0)	2 (16.6)	28 (58.3)	.01
Time in natalizumab until vaccination, mean (SD), mo	86.3 (56.1)	1.4 (3.2)	107 (40.8)	<.001
Disease duration, mean (SD), y^a^	16.7 (8.7)	7.7 (9.5)	18.9 (7.1)	<.001
Initiating treatment with natalizumab	8 (13.3)	6 (50.0)	2 (4.2)	<.001
Annualized relapse rate the year before index date, mean (SD)	0.28 (0.66)	1.41 (0.79)	0.00 (0.00)	<.001
Contrast enhancing lesions the year before index date, mean (SD)^b^	2.57 (4.23)	5.6 (4.75)	0.0(0.0)	<.001
New T2 the year before index date, mean (SD)	0.74 (3.16)	5.00 (8.00)	0.0 (0.14)	<.001
Expanded Disability Status Scale score at vaccination, median (IQR)	3.5 (2.0-6.0)	2.0 (1.5-2.5)	4.0 (2.5-6.0)	.001
John Cunningham Virus index at vaccination				
≤0.9	37 (61.6)	8 (66.6)	29 (60.4)	.54
1.0-1.5	3 (5.0)	1 (8.3)	2 (4.1)	.54
>1.5	20 (33.3)	3 (25.0)	17 (35.4)	.54
Type of vaccine administered				
Hepatitis A	24 (40.0)	5 (41.6)	19 (39.5)	>.99
Hepatitis B	27 (45.0)	8 (66.6)	19 (39.5)	.11
COVID-19	23 (38.0)	4 (33.3)	19 (39.5)	.75

^a^ First symptom to vaccination.

^b^ Information available for 26 patients.

The short-term group, compared with the long-term group, exhibited a greater mean (SD) ARR (1.41 [0.79] vs 0.00 [0.00]; *P* < .001), a greater mean (SD) NT2L (5.00 [8.00] vs 0.0 [0.14]; *P* < .001), and a greater mean (SD) number of contrast-enhancing lesions (5.6 [4.7] vs 0.0 [0.0]; *P* < .001), all of which indicate significantly higher disease activity for the short-term group. These observations aligned with our initial expectations.

Within the short-term group, the median (IQR) time between the last relapse and vaccination was 149 (112-207) days. Among the 12 patients in the short-term group, 8 received high doses of corticosteroids, with a median (IQR) duration of 95 (52-176) days between corticosteroid administration and vaccination. These parameters were not calculated for the long-term group due to the absence of relapses in the prevaccination period.

### Immunogenicity

**Figure 2.**
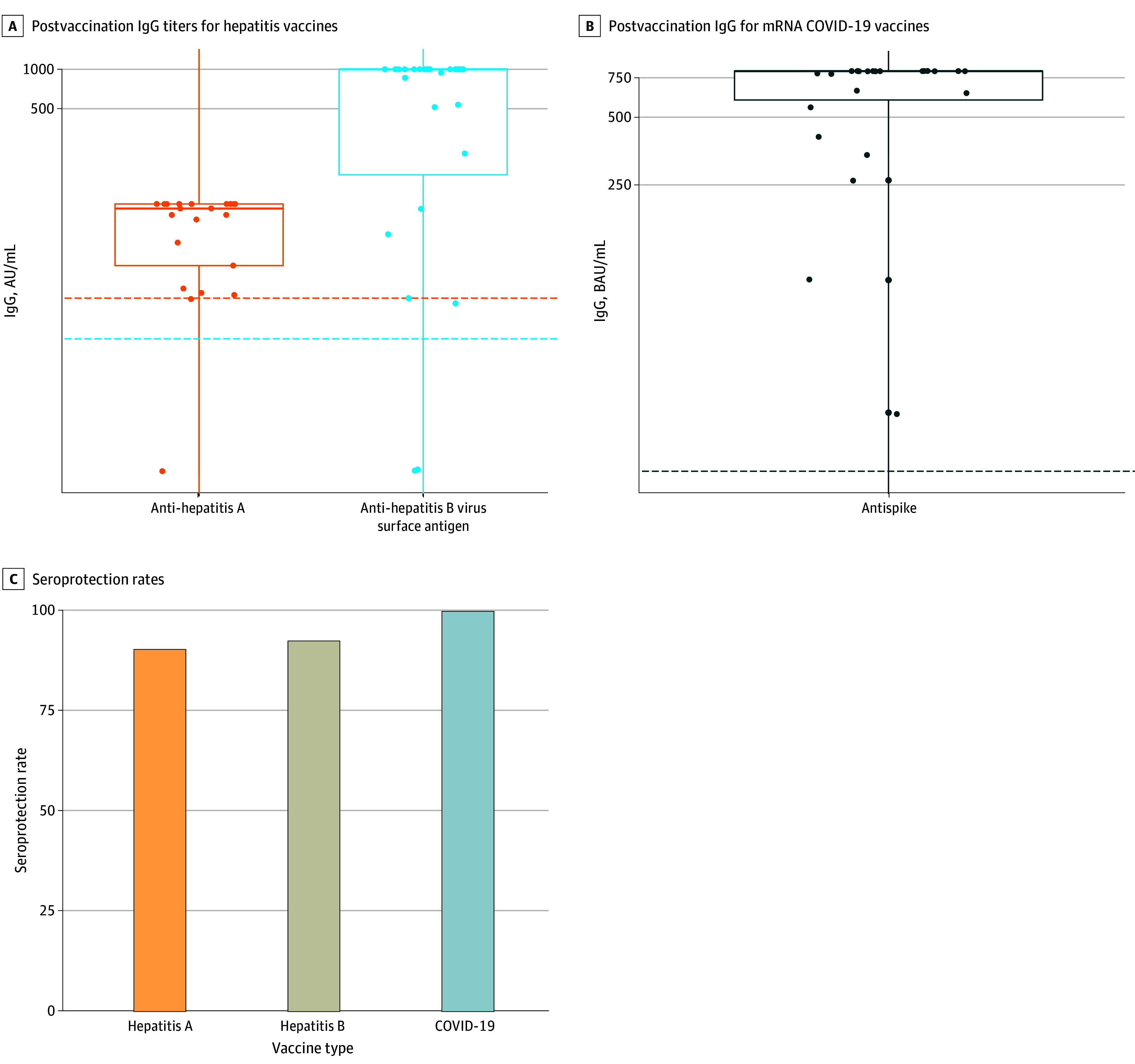
Immunogenicity of Vaccination in Patients With Multiple Sclerosis Treated with Natalizumab The figure shows immunoglobulin G (IgG) titers according to type of vaccine for hepatitis vaccines (A) and COVID-19 vaccines (B). Each dot in panels A and B represents a different participant. Cutoff values for antibody positivity are indicated by a dotted line. Panel C displays seroprotection rates for the hepatitis A, hepatitis B, and COVID-19 vaccines after vaccination. AU indicates antibody units; BAU, binding antibody units; mrNA, messenger RNA.

The overall seroprotection rate was 93% (95% CI, 86%-98%), with specific rates of 92% for HAV (95% CI, 73%-99%), 93% for HBV (95% CI, 76%-99%), and 100% for the mRNA COVID-19 vaccine (95% CI, 84%-100%). There was a substantial increase in postvaccination IgG titers, exceeding the cutoff level by more than 4-fold for all vaccines (Figure 2).

### Safety

**Figure 3.**
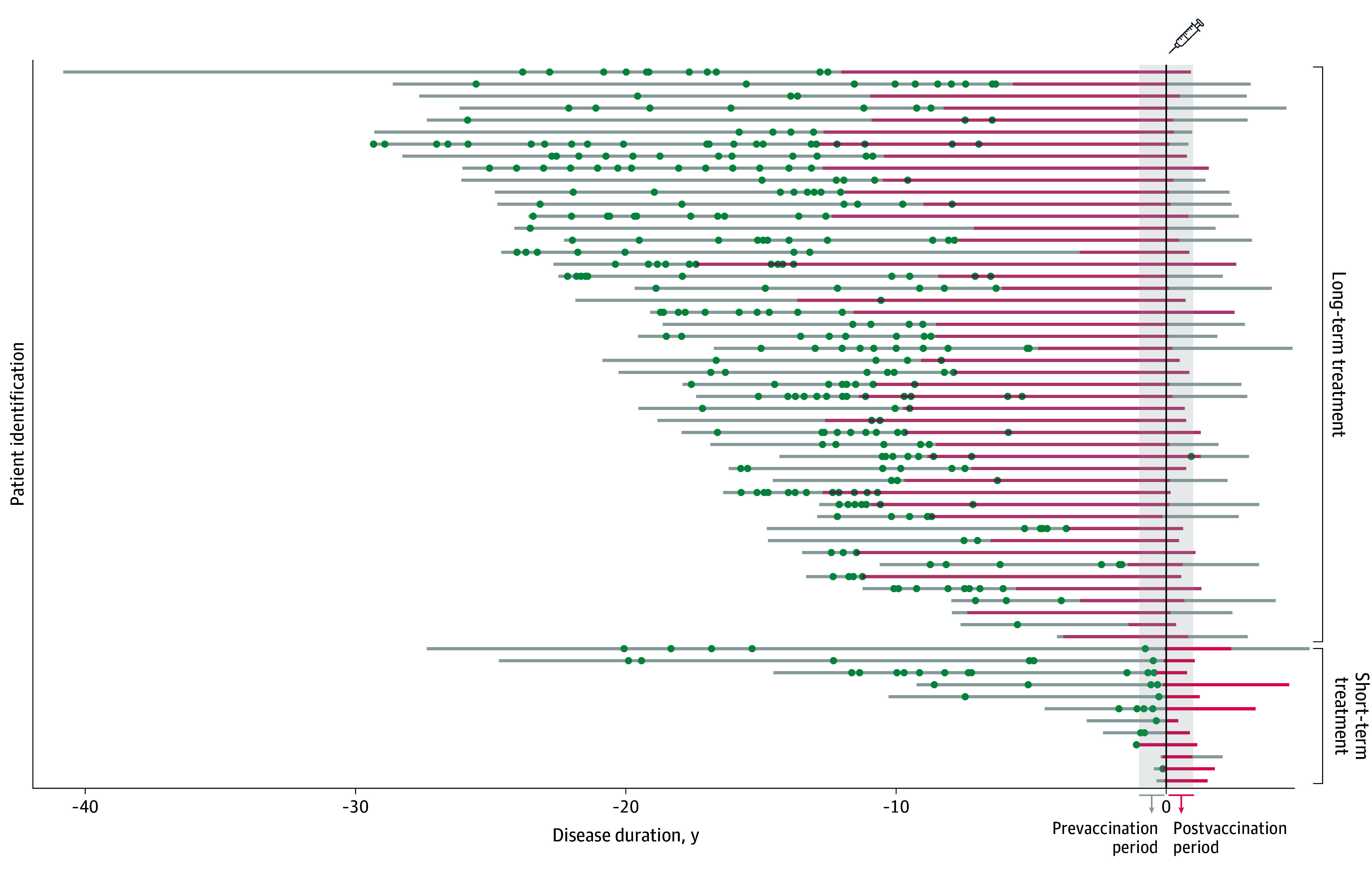
Disease Paths, Relapses, Natalizumab Exposure, and Differences Between Prevaccination and Postvaccination Periods Each line illustrates a patient’s journey from symptom onset to the latest follow-up. The line turns red during natalizumab treatment. Blue dots symbolize relapses. The shaded gray region marks the year before and after vaccination, corresponding to prevaccination and postvacciantion periods.

No worsening of clinical activity was observed between the prevaccination and postvaccination periods; in fact, an improvement was noted (mean [SD] prevaccination ARR 0.28 [0.66] vs. mean [SD] postvaccination ARR 0.01 [0.12]; *P* = .004). Only 1 patient experienced a single relapse in the postvaccination period, which occurred 338 days after vaccination (Figure 3). Importantly, no patients experienced relapses during the high-risk period. Additionally, no differences were noted between the prevaccination and postvaccination periods in terms of EDSS scores (median [IQR] score, 3.5 [2.0-6.0] vs 3.5 [2.0-6.0]; *P* = .62). Regarding MRI activity, none of the patients in the long-term natalizumab treatment group exhibited NT2L either before or after vaccination. Within the short-term group, no worsening of MRI activity was observed between the prevaccination and postvaccination periods. In fact, a significant reduction in median (IQR) NT2L was seen (5.00 [2.00-10.00] vs 0.81 [0.00-0.50]; *P* = .01). Only 3 patients in this group presented with NT2L in the postvaccination period, but the extent of lesions was significantly lower than in the prevaccination period. It is worth noting that these MRIs were performed shortly after natalizumab initiation (eTable 2 in Supplement 1).

### JCV Status

At the time of vaccination, 39 patients (65.0%) exhibited a positive JCV index, with 20 (51.2%) having high index values (>1.5). Remarkably, no cases of progressive multifocal leukoencephalopathy (PML) were observed in the treatment groups. Among the patients with a positive JCV index, 30 (79.0%) transitioned to anti-CD20 therapy after receiving their vaccination schedules. The mean (SD) time receiving natalizumab treatment for patients transitioning to anti-CD20 was 1.8 (0.9) years for the short-term exposure group and 8.9 (2.9) years for the long-term exposure group.

### Algorithm Development

**Figure 4.**
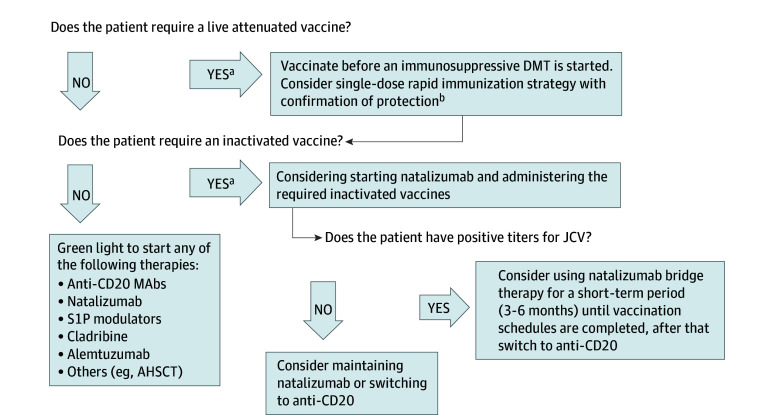
Proposed Algorithm for Immunizations in Patients With Highly Active Multiple Sclerosis Who Require Prompt Initiation of High Efficacy Disease Modifying Therapy^33,34^ AHSCT indicates autologous hematopoietic stem cell transplantation; DMT, disease-modifying therapy; JCV, John Cunningham virus; MAbs, monoclonal antibodies. ^a^According to the patients’ characteristics, background history of infections and vaccinations, serological tests, and past or future immunosuppressive therapies.^4,24,25^ ^b^For measles and varicella zoster virus.^35^

Based on our findings, we developed an immunization algorithm integrated into a risk-minimization strategy tailored for patients with highly active MS receiving natalizumab. This algorithm can be seen in Figure 4.

## Discussion

This cohort study assessed both the immunogenicity and safety of inactivated vaccines in PWMS receiving natalizumab. The seroprotection rates exceeded 90% for HAV, HBV, and mRNA COVID-19 vaccines, indicating an excellent response to immunization in PWMS undergoing natalizumab treatment. These findings were consistent with previous studies^10,11,12,13,14,15,16,36,37,38,39^ on other vaccines such as influenza, tetanus, diphtheria, and pertussis, providing new evidence for crucial vaccines such as HAV and HBV and reaffirming those for COVID-19 vaccines. Furthermore, all vaccines demonstrated a favorable safety profile, with no evidence of worsening disease activity in an important subgroup of patients with highly active MS, which had not, to our knowledge, been previously evaluated.

Until recently, the majority of data regarding vaccination in PWMS receiving highly effective drugs came from clinical trials and involved a limited number of patients, underscoring the need for more substantial empirical evidence.^10,40,41,42^ The COVID-19 pandemic was a turning point, and a large amount of data on vaccination has been published, adding considerable information on the safety and immunogenicity for the different vaccine formulations for this virus.^37,43,44,45,46^ The evidence coming from COVID-19 and non–COVID-19 vaccines consistently points to a decreased immune humoral response in PWMS treated with highly effective drugs, such as B-cell depleting therapies or S1P modulators.^40,41^ For these particular DMTs, the timing between vaccination and treatment initiation appears crucial for achieving optimal immunization with acceptable safety. As a result, current guidelines^24,25,47^ recommend administering inactivated vaccines at least 2 weeks before initiating treatment. However, some vaccine schedules require multiple doses administered at intervals (eg, the HBV schedule includes 3 or 4 doses with 6-month intervals), potentially causing delays in the initiation of treatment for highly active PWMS.^8,48^ HBV is strongly recommended for PWMS, especially those undergoing anti-CD20 therapy.^25,49^ In our study, a substantial proportion of patients who transitioned to anti-CD20 received the HBV vaccine, ensuring excellent seroprotection before the initiation of anti-CD20 therapy. The HAV vaccine, on the other hand, is strongly recommended for PWMS, especially international travelers who are seronegative, because hepatitis A in adults is a serious liver disease.^24,48,50^ Considering the excellent seroprotection rate and postvaccination IgG titers observed in our study, natalizumab may serve as a valuable therapeutic strategy to strike the right balance between providing early, highly effective treatment and achieving optimal immunization. This phenomenon could be attributed to the natalizumab antitraffic mechanism, which differs from the cell depletion observed in other agents.^51^

Furthermore, a pivotal aspect concerns the safety of vaccinations for PWMS. Some evidence has been debated, suggesting that certain vaccination schedules may potentially exacerbate MS activity.^52,53,54^ It is worth noting that only a limited number of studies^18^ have delved into this crucial matter concerning patients with highly active MS who are theoretically the most immunologically vulnerable to flare-ups. Moreover, this concern is amplified by a growing trend of people declining vaccinations due to safety concerns.^55^ Our study aimed to address this knowledge gap, revealing no elevated risk of clinical or radiological activity during the postvaccination period. This finding is particularly crucial for patients in the early stages of natalizumab treatment (ie, the short-term group), a critical period where higher disease activity might be anticipated, in comparison with those receiving longer natalizumab treatment (ie, the long-term group). Consistent with our findings, several studies^30,56,57,58^ have indicated that commonly administered live or inactivated vaccines do not increase the risk of exacerbations or disability progression in untreated PWMS. However, it is noteworthy that none of these studies included patients with highly active MS using immunosuppressive therapies. Recent evidence includes a publication from a large French registry,^59^ which found no association of vaccination with the risk of hospitalization due to MS relapse. Nevertheless, the proportion of patients with highly active MS treated with high-efficacy drugs was not described.

There are additional considerations for patients undergoing natalizumab treatment, such as the risk of PML and the potential for disease activity rebound after discontinuation of natalizumab. Postmarketing evidence^32,60^ has shown that the incidence of PML remains extremely low during the initial 12 months of treatment, regardless of the JCV index value. Considering that most recommended vaccination schedules for PWMS do not extend beyond 6 months,^24,25^ the utilization of natalizumab for a brief duration (3-6 months) in patients with a positive JCV index might be a viable option to contemplate. It is worth noting that approximately two-thirds of our vaccinated patients had a positive JCV index at the time of vaccination. Many of these patients subsequently transitioned to anti-CD20 therapy shortly after completing their immunization schedules. Importantly, these patients initiated or continued treatment with natalizumab and completed their vaccination regimen without experiencing any incidents of PML. Furthermore, it is noteworthy that no instances of disease activity rebound were observed in this study. This lack of rebound can be attributed to the swift transition of patients to alternative treatments with comparable efficacy profiles, such as anti-CD20 monoclonal antibodies.

Another aspect to consider is our decision to include inactivated HAV, HBV, and mRNA COVID-19 vaccines, which allowed us to evaluate various types of vaccine formulations (eg, whole virus, recombinant antigen, and mRNA technology). Furthermore, for the hepatitis vaccines, the availability of a prespecified serostatus cutoff enabled us to use it as a surrogate marker for assessing seroresponse.^27,28,29^

### Strengths and Limitations

This study has multiple strengths. First, it involved patients from an ongoing cohort, ensuring regular assessments following a well-defined protocol. This method yielded prospective documentation of high-quality clinical, radiological, treatment, serological, and vaccination data. Second, our study assessed both the immunogenicity and safety outcomes of vaccination in patients with highly active MS, addressing a critical aspect and filling an essential gap, as mentioned earlier. Lastly, we provided evidence regarding commonly used vaccines such as hepatitis vaccines and confirmed findings from previous COVID-19 studies.^16,37,39^

However, we also acknowledge some limitations. Despite the prospective data collection, obtaining comprehensive and precise retrospective information in an empirical context remains challenging. As a result, the total number of patients treated with natalizumab in each vaccine group was relatively low, precluding a separate safety analysis for each vaccine. Furthermore, we were only able to examine a limited number of vaccinated patients during the first year of natalizumab treatment (short-term group), specifically those who transitioned to anti-CD20 therapy as part of a bridge therapy strategy. Consequently, larger multicenter studies focusing on this subgroup will be needed. Another limitation is associated with the self-control analysis for the safety objective because this methodology may have limitations in terms of generalizability to the broader MS population. However, it is important to note that our study was specifically designed to ensure the safety of using inactivated vaccines for patients with highly active MS, for whom natalizumab is typically prescribed. In this context, the self-controlled cohort design proves particularly valuable for studying infrequent events, such as relapses associated with vaccination, as demonstrated in previous works addressing this aspect.^30,31,53^

## Conclusion

The findings of this cohort study suggest that immunization with inactivated vaccines such as HAV, HBV, and mRNA COVID-19 under natalizumab therapy was both safe and immunogenic, regardless of short-term or long-term treatment duration. These findings provide valuable empirical insights, indicating potential benefits for patients with highly active MS who require both immunization and high-efficacy therapies that might affect vaccine responses. In such scenarios, considering the use of natalizumab until vaccination schedules are successfully completed could be advantageous. Furthermore, based on JCV serostatus, maintaining natalizumab treatment or using it as a bridge therapy for a short-term period (3-6 months) in individuals with high titers may be a prudent approach. We propose an immunization algorithm integrated into a risk-minimization strategy tailored for patients with highly active MS, incorporating the findings from our study and emphasizing the pivotal role of natalizumab (Figure 4). This approach holds promise in averting treatment delays and providing adequate protection against potentially severe infections, particularly in the contemporary therapeutic landscape. Further investigations are needed to formally evaluate this strategy.

## Notes

Supplement 1. **eFigure.** Study Design **eTable 1.** Comparison (at the Time of Analysis) Between Vaccinated (Study Population) and Nonvaccinated Patients Receiving Natalizumab During the Specified Window Period **eTable 2.** Patients Exhibiting Inflammatory Activity During the Postvaccination Period

Supplement 2.
Data Sharing Statement
