# Supplementary material for: Vaccine Safety and Immunogenicity in Patients With Multiple Sclerosis Treated With Natalizumab
Source: JAMA Netw Open. 2024 Apr 12;7(4):e246345. doi: 10.1001/jamanetworkopen.2024.6345 (PMC11015356; doi:10.1001/jamanetworkopen.2024.6345)
Supplement: Supplement 1. — eFigure. Study Design eTable 1. Comparison (at the Time of Analysis) Between Vaccinated (Study Population) and Nonvaccinated Patients Receiving Natalizumab During the Specified Window Period eTable 2. Patients Exhibiting Inflammatory Activity During the Postvaccination Period [file jamanetwopen-e246345-s001.pdf]

Supplemental Online Content

Carvajal R, Zabalza A, Carbonell-Mirabent P, et al. Vaccine safety and immunogenicity in patients with multiple sclerosis treated with natalizumab. *JAMA Netw Open*. 2024;7(4):e246345. doi:10.1001/jamanetworkopen.2024.6345

eFigure. Study Design

eTable 1. Comparison (at the Time of Analysis) Between Vaccinated (Study Population) and Nonvaccinated Patients Receiving Natalizumab During the Specified Window Period

eTable 2. Patients Exhibiting Inflammatory Activity During the Postvaccination Period

This supplemental material has been provided by the authors to give readers additional information about their work.

© 2024 Carvajal et al. *JAMA Network Open.*

eFigure. Study Design

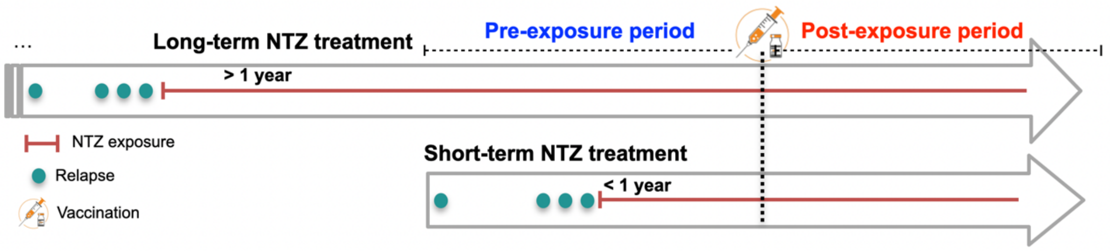

Abbreviations: NTZ = Natalizumab

eTable 2. Patients Exhibiting Inflammatory Activity During the Postvaccination Period

	Disease activity	Vaccination date	NTZ onset	Date of relapse or NT2L[a]	Distance between vaccination and disease activity	NTZ group	Inflammatory activity the previous year to vaccination
**1**	1 relapse	22/08/2019	22/10/2010	26/07/2020	338 days	Long	No
**2**	5 NTL2	22/10/2020	22/10/2020	30/12/2020 vs 15/07/2020	68 days	Short	1 relapse, 12 CELs
**3**	3 NTL2	16/07/2020	29/05/2020	27/11/2020 vs 18/05/2020	123 days	Short	1 relapse, 5 CELs
**4**	1 NTL2	30/01/2019	14/12/2018	19/11/2019 vs 18/09/2018	376 days	Short	1 relapse, 5 CELS

[a.] Date of the MRI where inflammatory activity was documented and the date of the comparative MRI.
Abbreviations: NTZ = Natalizumab. NT2L = New T2 lesions. CELs = Contrasts-enhancing lesions.

© 2024 Carvajal et al. *JAMA Network Open.*

eTable 1. Comparison (at the Time of Analysis) Between Vaccinated (Study Population) and Nonvaccinated Patients Receiving Natalizumab During the Specified Window Period

General characteristics	Vaccinated during NTZ (n=60)	No vaccinated during NTZ (n=167)	p
Age, M(SD)	45.1 (9.4)	41 (10.6)	.01
Female n(%)	44 (73.3)	109 (65.2)	.32
Change NTZ to anti CD20 n (%)	32 (53.3)	69 (41.3)	.14
Time in NTZ (months), M(SD)	97.1 (53.9)	72.3 (56.7)	.003
Disease duration (yr)*, M (SD)	19.2 (8.9)	15.9 (8.7)	.01

Abbreviations: SD= Standard deviation. NTZ = Natalizumab.

© 2024 Carvajal et al. *JAMA Network Open.*
