# Supplementary material for: Vaccine Safety and Immunogenicity in Patients With Multiple Sclerosis Treated With Natalizumab
Source: JAMA Netw Open. 2024 Apr 12;7(4):e246345. doi: 10.1001/jamanetworkopen.2024.6345 (PMC11015356; doi:10.1001/jamanetworkopen.2024.6345)
Supplement: Supplement 2. — Data Sharing Statement [file jamanetwopen-e246345-s002.pdf]

Data Sharing Statement

Carvajal. Vaccine Safety and Immunogenicity in Patients With Multiple Sclerosis Treated With Natalizumab. *JAMA Netw Open*. Published April 12, 2024.
doi:10.1001/jamanetworkopen.2024.6345

Data
Data available: Yes
Data types: Deidentified participant data
How to access data: rcarvajal@cem-cat.org
When available: With publication

Supporting Documents
Document types: None

Additional Information
Who can access the data: Anyone requesting the data, however, all requests will need to explain in detail the hypothesis of the study that the researchers want to carry out and the analysis plan.
Types of analyses: For a specific purpose, the appropriateness of data sharing in each particular case will be thoroughly discussed in ad hoc research meetings.
Mechanisms of data availability: If the decision is that data will be shared, all applicants will be asked to sign a data access agreement.
